# Differences in mtDNA haplogroup distribution among 3 Jewish populations alter susceptibility to T2DM complications

**DOI:** 10.1186/1471-2164-9-198

**Published:** 2008-04-29

**Authors:** Jeanette Feder, Ilana Blech, Ofer Ovadia, Shirly Amar, Julio Wainstein, Itamar Raz, Sarah Dadon, Dan E Arking, Benjamin Glaser, Dan Mishmar

**Affiliations:** 1 Department of Life Sciences and National Institute of Biotechnology in the Negev (NIBN), Ben-Gurion University of the Negev, Beer-Sheva, Israel; 2 Endocrinology and Metabolism Service, Internal Medicine Department, Hadassah-Hebrew University Medical Center, Jerusalem, Israel; 3 Stanley Research Center, Ben-Gurion University of the Negev and Mental Health Center, Beer-Sheva, Israel; 4 Israel Diabetes Research Group (IDRG), Israel; 5 Wolfson Hospital, Holon, Israel; 6 Diabetes Unit, Internal Medicine Department, Hadassah-Hebrew University Medical Center, Jerusalem, Israel; 7 McKusick-Nathans Institute of Genetic Medicine, Johns Hopkins University School of Medicine, Baltimore, Maryland 21205, USA

## Abstract

**Background:**

Recent genome-wide association studies searching for candidate susceptibility loci for common complex diseases such as type 2 diabetes mellitus (T2DM) and its common complications have uncovered novel disease-associated genes. Nevertheless these large-scale population screens often overlook the tremendous variation in the mitochondrial genome (mtDNA) and its involvement in complex disorders.

**Results:**

We have analyzed the mitochondrial DNA (mtDNA) genetic variability in Ashkenazi (Ash), Sephardic (Seph) and North African (NAF) Jewish populations (total n = 1179). Our analysis showed significant differences (p < 0.001) in the distribution of mtDNA genetic backgrounds (haplogroups) among the studied populations. To test whether these differences alter the pattern of disease susceptibility, we have screened our three Jewish populations for an association of mtDNA genetic haplogroups with T2DM complications. Our results identified population-specific susceptibility factors of which the best example is the Ashkenazi Jewish specific haplogroup N1b1, having an apparent protective effect against T2DM complications in Ash (p = 0.006), being absent in the NAF population and under-represented in the Seph population. We have generated and analyzed whole mtDNA sequences from the disease associated haplogroups revealing mutations in highly conserved positions that are good candidates to explain the phenotypic effect of these genetic backgrounds.

**Conclusion:**

Our findings support the possibility that recent bottleneck events leading to over-representation of minor mtDNA alleles in specific genetic isolates, could result in population-specific susceptibility loci to complex disorders.

## Background

The quest for susceptibility genes of common complex disorders such as type 2 diabetes mellitus (T2DM) has led to recent successful discoveries of novel disease-related genes through the use of large scale genome-wide association studies including thousands of patients belonging to major ethnic groups [1]. Disease-associated loci often fail to replicate in different populations, because of patterns of population-specific susceptibility [2]. This may occur due to genetic drift and founder effects, turning minor alleles in a certain populations to prevalent ones in another population. One may hypothesize that some of these alleles carry functional effects underlying differences in disease susceptibility between populations. Revealing such an effect requires mining special populations, such as the Jews, that due to bottleneck events have increased incidence of alleles that are less abundant in the general population.

The Jewish people underwent several recent bottleneck events after the 2600 year old Babylonian and 2000 year old Roman deportation from Israel [3,4]. These resulted in geographically separated Jewish communities that kept their customs and religion over centuries, mostly marrying within the communities with little or no intermarriage with local non-Jews, suggesting several founder events. Thus, Jews represent an excellent model to study possible association of population-specific alleles with common disorders, including T2DM [5].

T2DM is the most common metabolic disease today, with increasing incidence in the Western world (1). Growing evidence for dysfunction of the mitochondrial energy production machinery (OXPHOS) in many T2DM patients [6] highlights the role of altered OXPHOS activity in the molecular basis leading to the common forms of T2DM: (a) approximately 1% of diabetic patients have large mitochondrial DNA (mtDNA) deletions or the A3243G point mutation [7,8]; (b) expression of OXPHOS-related genes is decreased in muscle tissues of diabetic individuals [9,10]; (c) mitochondrial ATP production is decreased and intra-myocellular fat content is increased in offspring of T2DM patients [11]; (d) in pancreatic beta-cells of mice, cellular depletion of mtDNA and knock-out of mitochondrial transcription factor A (TFAM) interfere with insulin secretion [12,13].

Given that T2DM is a common complex disorder with considerable heritability, it is probably influenced by a combination of predisposing common genetic variants, potentially including mtDNA variants. Although mtDNA genetic variants have previously been associated with complex disorders in some populations [14], its extensive genetic variability [15] and uniparental inheritance may result in diverse association among specific populations [16]. Indeed, mtDNA genetic association with T2DM exemplifies the differences among populations: significant association of certain mtDNA genetic backgrounds (haplogroups) was found in Asians [17] but not in Caucasians as documented in a recent large scale analysis [18]. Additionally, association of mtDNA variants with T2DM was limited to specific populations [19-21]. The only example of a mtDNA variant (T16189C) associated with T2DM in both Caucasian and Chinese populations [22,23] was recently questioned [24].

Similar to T2DM, diabetic complications are complex phenotypes determined by multiple pathways with a large genetic component. Diabetic complications increase markedly in incidence after 5–10 years of active T2DM, but with extreme variability in onset and progression, i.e. some individuals developing severe complications relatively early in the disease course, while others fail to develop any significant complications despite many years of severe disease. Being responsible for most T2DM-associated mortality, diabetic complications involve pathology in small and large vessels (micro- and macrovascular disease), encompassing malfunction of the mitochondrial OXPHOS [25]. Thus, mtDNA variants could be logical candidates to alter the genetic risk to the major diabetic complications- nephropathy, retinopathy and cardiovascular disease [26].

To search for possible population-specific association between mtDNA common genetic variants and the common complications of T2DM we examined mtDNA genetic variability in three Jewish populations: Ashkenazi, Sephardic and North African Jews.

## Results

**Figure 1:**
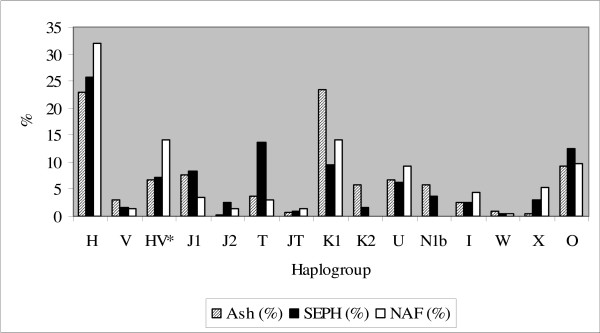
**Haplogroup distribution in the three studied populations: Letters under the X-axis – haplogroup names; Y-axis – percentage of each haplogroup of the corresponding populations (Ash – lines, Seph – black, NAF – white).** Ash (n = 762), Seph (n = 191), NAF (n = 226). HV* – samples belonging to the HV lineage, excluding haplogroups H and V. O – others; JT – samples belonging to the JT lineage excluding haplogroups J and T.

A total of 1,179 T2DM patients comprised of three populations (762 Ashkenazi Jews [Ash], 191 non-Ashkenazi European Jews [Seph], and 226 North African Jews [NAF]), were genotyped and assigned to different mtDNA haplogroups. Almost 90% of the subjects belonged to one of the 12 most prevalent mtDNA haplogroups in Ashkenazi Jews, i.e., K1, K2, U (non-K), H, V, J1, J2, T, N1b, I, X, W (Figure 1). R × C test of independence [27] indicated that haplogroup distribution varied significantly among the three Jewish patients populations (G = 180.1, df = 30, p < 0.001), implying that each of these patients populations should be analyzed separately. Specifically, there was an over-representation of haplogroup T in Seph patients, HV* in NAF patients, haplogroups K1, K2 and N1b in the Ashkenazi patients. Although the observed differences are among populations of patients and do not necessarily apply to the general population, our data is in line with previous reports of differences in mtDNA haplotypes distribution among Jewish populations, thus supporting the hypothesis that separate founder events led to the establishment these different Jewish populations (Figure 1) [3,28]. Furthermore, sequencing of the mtDNA hyper variable region 1 (HVR1) of haplogroup N1b patients in our populations revealed that the Ashkenazi population harbored only the 16145-16176A-16223 motif termed "N1b1" which is extremely rare in other populations, whereas of the seven Seph N1b patients, five harbored the N1b1 motif and two harbored a 16145-16176G-16223 motif termed "N1b2" which is found at low prevalence in Caucasians. Haplogroup N1b was totally absent from the NAF population, thus further supporting separate bottleneck events in the Ash, Seph and NAF populations.

This significant genetic divergence of mtDNA genetic variation could result in population-specific signals of mtDNA association with complex disorders. In order to evaluate this we have assessed possible association of mtDNA haplogroups with the major complications of T2DM separately within each of the three populations. This approach enabled minimizing the possible effects of population stratification. First, each population was divided into patients that had developed cardiovascular disease, retinopathy or nephropathy (Tables 1, 2, 3) and a group of patients who did not develop any of these complications after at least 10 years of known diabetes ("no-complications" group). These complications were chosen because of their high prevalence in T2DM and since the organs involved (heart, retina and kidney, respectively) are highly affected in mitochondrial disorders [29].

**Table 1:** Haplogroup distribution of Ashkenazi (Ash) T2DM patients who developed complications and those who did not develop any complications for at least 10 years after diagnosis ("No complications"). Patients included in this table may have more than one complication. N – number of patients in each group; (%) – percentage of patients belonging to a certain haplogroup in a specific group.

**Haplogroup**	**All Ash N (%)**	**No complications N (%)**	**Cardiovascular N (%)**	**Retino-pathy N (%)**	**Nephro-pathy N (%)**
**U (nonK)**	**51 (6.7)**	**19 (7.1)**	**12 (4.6)**	**9 (7.6)**	**22 (7.1)**
K1	178 (23.4)	56 (21)	65 (24.9)	25 (21.2)	77 (25)
**K2**	**44 (5.8)**	**17 (6.4)**	**14 (5.4)**	**6 (5.1)**	**19 (6.2)**
^1^HV*	74 (9.7)	26 (9.7)	27 (10.3)	14 (11.9)	25 (8.1)
**H**	**175 (23)**	**60 (22.5)**	**66 (25.3)**	**31 (26.3)**	**73 (23.7)**
J1	59 (7.8)	14 (5.2)	18 (6.9)	14 (11.9)	33 (10.7)
**T**	**33 (4.3)**	**13 (4.9)**	**9 (3.4)**	**3 (2.5)**	**12 (3.9)**
N1b	44 (5.8)	23 (8.6)	13 (5)	6 (5.1)	9 (2.9)
**IWX**	**31 (4.1)**	**12 (4.5)**	**8 (3.1)**	**2 (1.7)**	**16 (5.2)**
^2^Others	71 (9.3)	27 (10.1)	29 (11.1)	8 (6.8)	22 (7.1)
**Total**	760**	267**	**261**	**118**	**308**

^1^HV* – samples belonging to the HV lineage, excluding haplogroup H but including haplogroup V. ^2^Others – include haplogroups JT* and L in addition to unclassified, probably non-European haplogroups. **After removing the two J2 patients (see Materials and Methods), we were left only with J1 patients; thus, the sample size of the "no-complications" group was reduced from 269 to 267, and that of the total population from 762 to 760.

**Table 2:** Haplogroup distribution of European non-Ashkenazi Jewish (Seph) T2DM patients who developed complications and the "No complications" group as in table 1. Patients included in this table may have more than one complication.

**Haplogroup**	**All Seph n (%)**	**No complication n (%)**	**Cardiovascular n (%)**	**Retino-pathy n (%)**	**Nephro-pathy n (%)**
U (nonK)	12 (6.5)	5 (7.7)	3 (5.4)	1 (2.7)	5 (7.7)
**K**	**21 (11.3)**	**6 (9.2)**	**7 (12.5)**	**7 (18.9)**	**9 (13.8)**
^1^HV*	17 (9.1)	4 (6.2)	5 (8.9)	7 (18.9)	10 (15.4)
**H**	**49 (26.3)**	**17 (26.2)**	**16 (28.6)**	**6 (16.2)**	**14 (21.5)**
J1**	16 (8.6)	5 (7.7)	3 (5.4)	4 (10.8)	8 (12.3)
**T**	**26 (14)**	**12 (18.5)**	**7 (12.5)**	**4 (10.8)**	**4 (6.2)**
N1b	7 (3.8)	2 (3.1)	2 (3.6)	2 (5.4)	3 (4.6)
**WXI**	**12 (6.5)**	**4 (6.2)**	**3 (5.4)**	**3 (8.1)**	**3 (4.6)**
^2^Others	26 (14)	10 (15.4)	10 (17.6)	3 (8.1)	9 (13.8)
**Total**	**186**	**65**	**56**	**37**	**65**

^1^HV* – see table 1. ^2^Others – see table 1. **After removing the J2 patients (see Materials and Methods), we were left only with J1 patients; thus, the sample size was reduced from 191 to 186 for the total population and the complications groups were adjusted accordingly.

**Table 3:** Haplogroup distribution of North African Jewish (NAF) T2DM patients who developed complications and the "No complications" group as in table 1. Patients included in this table may have more than one complication.

**Haplogroup**	**All NAF n (%)**	**No complication n (%)**	**Cardiovascular n (%)**	**Retino-pathy n (%)**	**Nephro-pathy n (%)**
**U (nonK)**	**21 (9.4)**	**9 (12.3)**	**5 (9.8)**	**3 (5)**	**8 (9.1)**
K	32 (14.3)	12 (16.4)	4 (7.8)	10 (16.7)	9 (10.2)
^1^HV*	**35 (15.7)**	**6 (8.2)**	**13 (25.4)**	**12 (20)**	**19 (21.6)**
H	72 (32.3)	20 (27.4)	20 (39.2)	22 (36.7)	30 (34.1)
J1**	**8 (3.6)**	**5 (6.8)**	**1 (2)**	**0**	**3 (3.4)**
T	7 (3.1)	4 (5.5)	1 (2)	1 (1.7)	2 (2.3)
**WXI**	**23(10.3)**	**9 (12.3)**	**4 (7.8)**	**4 (6.7)**	**8 (9.1)**
^2^Others	25 (11.2)	8 (11)	3 (5.9)	8 (13.3)	9 (10.2)
**Total**	**223**	**73**	**51**	**60**	**88**

^1^HV* – see table 1.^2^Others – see table 1. **After removing the J2 patients (see Materials and Methods), we were left only with J1 patients; thus, the sample size was reduced from 226 to 223 for the total population and the complications groups were adjusted accordingly.

In an attempt to identify candidate haplogroups for association with T2DM complications a permutation analysis was performed (see Additional file 1 and Additional file 1 – Table 1). In the Ash population haplogroup J1 was detected as a plausible candidate for association with retinopathy and nephropathy (p = 0.035 and p = 0.022, respectively) and haplogroup N1b1 for association with nephropathy (p = 0.003) (Table 1). In the Seph population haplogroup aggregate HV* and haplogroup T were detected as borderline candidates for association with retinopathy (p = 0.054) and nephropathy (p = 0.059), respectively (Table 2). In the NAF population haplogroup aggregate HV* was detected as a candidate for association with nephropathy and cardiovascular disease (p = 0.024 and p = 0.014, respectively) (Table 3).

These results suggest that the differences in haplogroup distribution may result in different disease-associated mtDNA factors in each population. To rigorously investigate the involvement of mtDNA haplogroups in the tendency to develop T2DM complications we focused only on the significant candidate haplogroups (J1 and N1b in the Ash population, and HV* in the NAF population).

### mtDNA Haplogroups J1 and N1b associate with T2DM Complications in Ashkenazi Jews

Using a logistic regression model and appropriate Bonferonni correction we compared the candidate haplogroups with each of the other haplogroups while controlling for the possible effects of patient characteristics (disease duration, sex and age). A possible association of a population specific mtDNA haplogroup with T2DM complications could be best tested in our Ashkenazi population (Ash), since N1b1 is an apparently Ashkenazi-specific haplogroup. Our analyses revealed that haplogroup N1b was significantly under-represented in the nephropathy group and in the cardiovascular group as compared with the no-complication group relative to all other haplogroups (p = 0.006, odds ratio (OR) = 0.34 (0.15–0.74), and p = 0.017, OR = 0.39 (0.18–0.84), respectively; also see Additional file 1 – Table 2). In the retinopathy group however, no significant association with N1b was found. These results imply that Ashkenazi T2DM patients pertaining to haplogroup N1b exhibit reduced susceptibility to the tested T2DM complications.

In contrast to haplogroup N1b, haplogroup J1 was over-represented in the Ash population only in the microvascular complications (retinopathy and nephropathy). A significant and specific effect of haplogroup J1 could be masked by including patients who exhibit more than one complication in each of the tested groups. This premise is supported by the view that the risks of developing each of the different complications are not entirely independent. Retinopathy and nephropathy may have some common pathophysiological features, and nephropathy per se may increase the risk of cardiovascular disease. Hence reciprocal interactions among the complications could mask the effect of certain genetic backgrounds. To test for the possibility that patients pertaining to mitochondrial haplogroup J1 exhibit preferentially altered susceptibility to either microvascular or macrovascular complications, we performed the analysis on groups of T2DM patients having nephropathy or retinopathy but no evidence of cardiovascular disease and on T2DM patients with cardiovascular disease who did not develop either nephropathy or retinopathy (for detailed information of haplogroup J1 compared to each of the other haplogroups, see Additional file 1 – Table 3). This demonstrated a significant over-representation of haplogroup J1 in the "isolated" nephropathy group [p = 0.018, OR = 2.3 (1.15–4.7)] and in the "isolated" retinopathy group [p = 0.017, OR = 3.1 (1.2–7.8)]. However, no such trends could be detected in the "isolated" cardiovascular group. These results suggest that mutations defining mtDNA haplogroup J1 increase the propensity of Ashkenazi T2DM patients to develop nephropathy or retinopathy but not cardiovascular complications.

### mtDNA Haplogroups Associate with T2DM Complications in Non-Ashkenazi Jews

In accordance with our permutation test, logistic regression analysis revealed that NAF patients pertaining to the HV* haplogroup aggregate had a threefold risk of developing nephropathy [p = 0.027, OR = 3.12 (1.14–8.6)] and a fourfold risk of developing cardiovascular disease [p = 0.014, OR = 4.13 (1.33–12.9)] (see Additional file 1 – Table 4). Notably our permutation test showed the same tendency towards over-representation of haplogroup HV* in Seph patients with nephropathy (Table 4) with borderline significance (see Additional file 1 – Table 1).

**Table 4:** General clinical data for the patient populations.

**Parameter**	**Population**	**Total populations**	**No complications**	**Nephro-pathy**	**Retino-pathy**	**Cardiovascular**
Number of patients (% of total population)	Ash	762	269 (35.3)	308 (40.4)	118 (15.5)	261 (34.3)
	Seph	191	65 (34)	69 (36.1)	37 (19.4)	60 (31.4)
	NAF	226	74 (32.7)	88 (38.9)	61 (27)	52 (23)
**M/F (%)**	**Ash**	**50/50**	**38/62**	**54/46**	**57/43**	**62/38**
	**Seph**	**51/49**	**48/52**	**55/45**	**54/46**	**62/38**
	**NAF**	**48/52**	**42/58**	**53/47**	**48/52**	**63.5/36.5**
Age (mean ± SD)	Ash	65.7 ± 9.9	64.3 ± 10	65.9 ± 9.7	64.6 ± 9.2	68.0 ± 9.2
	Seph	65 ± 9	64.3 ± 8.4	65.1 ± 8.8	64.6 ± 8.2	66.8 ± 8.6
	NAF	61.9 ± 9.4	60.7 ± 10.0	62.4 ± 8.9	61.6 ± 10.3	63.1 ± 8.8
**Years of diabetes (mean ± SD)**	**Ash**	**18.9 ± 8.3**	**16.8 ± 7.3**	**19.2 ± 8.4**	**22.9 ± 8.5**	**20.9 ± 9.0**
	**Seph**	**19.4 ± 8.4**	**21.4 ± 8.8**	**20.5 ± 9.2**	**24 ± 9.1**	**21.2 ± 8.2**
	**NAF**	**19 ± 7.3**	**16.8 ± 6.4**	**19.4 ± 7.3**	**22.4 ± 7.9**	**19.7 ± 7.9**
BMI^1 ^(kg/m^2^, mean ± SD)	Ash	29.9 ± 5.2	28.8 ± 4.6	30.6 ± 5.5	30.8 ± 6.0	29.7 ± 5.0
	Seph	30.3 ± 4.9	30.7 ± 5.4	30.4 ± 5.3	30.5 ± 5.7	30.5 ± 5
	NAF	29.6 ± 5.1	28.8 ± 4.8	30.6 ± 5.1	30.3 ± 5.3	30.9 ± 4.5
**HbA1c (mean ± SD)**	**Ash**	**7.9 ± 1.5**	**7.8 ± 1.4**	**8.0 ± 1.5**	**8.2 ± 1.6**	**7.9 ± 1.4**
	**Seph**	**8.2 ± 1.6**	**8.2 ± 1.7**	**8.2 ± 1.4**	**8.2 ± 1.6**	**8.3 ± 1.6**
	**NAF**	**8.7 ± 1.9**	**8.7 ± 2.0**	**8.8 ± 2.0**	**8.6 ± 1.6**	**8.7 ± 1.9**
HTN^2 ^(%)	Ash	72.9	61.3	81.8	79.7	77.4
	Seph	70.2	73.8	81.2	86.5	81.7
	NAF	61.5	54.1	69.3	72.1	65.4
**LDL-c (mg/dl, mean ± SD)**	**Ash**	**104.0 ± 31.0**	**108.7 ± 31.1**	**102.3 ± 29.7**	**103.3 ± 27.9**	**98.2 ± 30.5**
	**Seph**	**109.3 ± 36.7**	**106 ± 36**	**107.1 ± 39**	**110.9 ± 38**	**96.1 ± 29.4**
	**NAF**	**104.7 ± 29.8**	**104.9 ± 28.3**	**106.3 ± 27.8**	**105.6 ± 36.6**	**102.5 ± 31**

^1 ^BMI = Body mass index ^2 ^HTN – Hypertension

Taken together these observations suggest that different mtDNA haplogroups may play a role in the propensity of Jewish T2DM patients to develop complications in the studied populations and that this propensity may be population specific.

### Evaluating the Functional Significance of Mutations Defining Haplogroups N1b and J1

**Figure 2:**
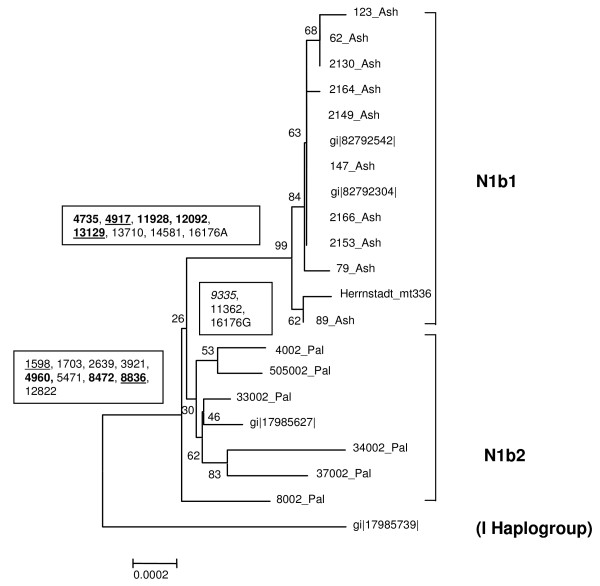
**Neighbor joining phylogenetic reconstruction of whole mtDNA sequences of the N1b haplogroup.** Haplogroup I sequence was used as an out-group, since it is the phylogeneticaly closest haplogroup to N1b. Sequences were aligned and bootstrapped 1000 times, and the tree was built with MEGA3 software. Sequence names ending with either "Ash" or "Pal" were generated by us indicating non-diabetic Ashkenazi Jews and Israeli Arabs, respectively; otherwise a Genbank Identification number (GI) was mentioned. It is worth noting that gi|82792542 and gi|82792304 N1b1 sequences originate from Ashkenazi Jews whereas the N1b2 sequence gi|17985627 is of non Jewish Jordanian origin. Sequence designated Herrnstadt2002-336 was downloaded from [45], as it was not available from Genbank. Numbers near the branches are the bootstrap values; numbers in boxes are changes in nucleotide positions of the mtDNA in the relevant node. Bold – amino acid change; underlined – change in a highly conserved position. For detailed information on each mutation and its degree of conservation, see Additional file 1 – Tables 5 and 6.

Extensive study has shown several mutations affecting nucleotide positions with a high degree of evolutionary conservation in haplogroup J1, possibly underlying their phenotypic consequences (see Discussion) [30,31]. Far less attention has been devoted to haplogroup N1b, although a recent study did indicate a difference between N1b sequences of Ashkenazi Jewish origin and N1b sequences in other groups [4]. To decipher the mutations underlying the phenotypic consequences of haplogroup N1b in the Ash population, we analyzed 20 whole N1b mtDNA sequences, 12 of which from Ashkenazi Jews, seven from other Middle-Eastern populations and one from a population of European origin. Sequence alignment and phylogenetic neighbor joining tree reconstruction, including a haplogroup I sequence as an out-group, revealed that sequences of Ashkenazi Jewish origin form a branch distinct from those of other Middle-Eastern populations (Figure 2). A close investigation of the sequences revealed nine coding region mutations in the stem of haplogroup N1b and additional eight mutations in the Ashkenazi N1b tree node (designated N1b1), whereas only three mutations lead to the non-Jewish tree node (designated N1b2). The N1b1 node harbors five amino acid changes in addition to the three amino-acid changes in the stem of the haplogroup. None of the changes in the N1b2 node alters an amino acid (Figure 2).

To test for the potential of the N1b-haplogroup-defining changes to alter function, we studied their degree of evolutionary conservation by investigating the alignment of mtDNA gene sequences from 42 different vertebrates and invertebrates (see Methods and Additional file 1 – Tables 5 and 6). The degree of conservation was ranked high only if it fell within one standard deviation range from the mean conservation degree of mtDNA disease-causing mutations [31] (Figure 2, Additional file 1 – Table 5). Strikingly, only the N1b1 node (Ashkenazi Jewish) holds highly conserved changes in addition to three highly conserved changes in the N1b stem thus supporting their potential involvement in the protective effect of haplogroup N1b1.

## Discussion

MtDNA genes, in contrast to nuclear DNA (nDNA)-encoded genes, are in full linkage disequilibrium. The mutation rate of the mtDNA is ~10 times faster than that of the nDNA and thus it is the most variable coding region in the human genome. Since mtDNA is maternally inherited, it is prone to genetic drift, resulting in large differences in patterns of genetic variability among and within populations [32]. Such genetic drift often leads to difficulties in replicating results of mtDNA association studies among populations. Hence, we hypothesized that, due to its high genetic divergence among populations, a subset of mtDNA alleles with functional consequences will differentiate among distinct populations. Here we have shown that recent bottleneck events within the three studied Jewish populations (Ash, Seph and NAF), underlie marked differences in mtDNA diversity in three ethnically-related Jewish populations, resulting in increased frequency of genotypes in some populations, some of which may act as susceptibility factors to T2DM complications. Such was the case in the haplogroup N1b1 that was significantly under-represented in certain complications of the Ashkenazi population and not present in the NAF population.

In contrast to haplogroup N1b1, the haplogroups identified as factors with risk trends to some T2DM complications (haplogroup J1 and haplogroup aggregate HV*, with marginal significant values considering a Bonferonni corrected α<0.017) were present in all three studied populations. Nevertheless haplogroup J1, showing association with increased risk for T2DM nephropathy or retinopathy in the Ashkenazi population does not have enough power to replicate in the Seph and NAF populations, i.e. ~250 subjects in each of the complications groups to replicate the significant results of the Ash population (power of 80%, α<0.05 (two tailed)) (Figure 1). The significant over-representation of haplogroup aggregate HV* in certain complications of the NAF population is harder to interpret, since although there was enough power to detect its effect in the Ashkenazi population, i.e. ~70 subjects in each of the complication groups in order to detect significance with a power of 80% and α<0.05 (two tailed), it did not show the same tendency as in the NAF population. Nevertheless one should take into account that HV* is a haplogroup aggregate; hence the different bottleneck events leading to the establishment of the Ashkenazi and North African Jewish populations could result in different compositions of lineages comprising the HV* haplogroup aggregate in the two populations. Testing for this possibility needs further genotyping of HV*, requiring increased sample sizes of the studied populations.

Our findings support association of mtDNA common genetic variants with sub-phenotypes of T2DM. Interestingly, the inconsistency of mtDNA genetic association with complications of T2DM as found here was described for other phenotypes as well: While haplogroup J has been associated with successful longevity in northern Italians and the Finnish [33], it was not associated with successful longevity in southern Italians [34] and subhaplogroup J1, but not J2, was associated with successful longevity in the Northern Irish [35]. Therefore, both differences in mtDNA sub-groups and their differences in response to environment appear to affect the relationship between mtDNA genotypes and phenotypes. Since all of the functional SNPs in particular mtDNA lineages would have a collective effect on mitochondrial function, many mtDNA haplogroups and sub-haplogroups might interact with environmental variation differently. Furthermore, this difference can be further complicated by the interaction of mtDNA encoded subunits, harboring functional SNPs, with nuclear DNA encoded subunits, harboring their own genetic variation. This interpretation applies to our observation that N1b1 reduces the risk to T2DM complications only in Ashkenazi Jews. In addition, the tendency to develop complications is an interplay of environment and genetics, hence it is not solely dependent on a particular haplogroup, and thus the absence of the N1b1 haplogroup in the NAF cohort is not expected to change significantly the overall risk do develop complications.

Since our study observed disease-association with a population specific haplogroup, the Ashkenazi specific N1b1, it was of importance to assess the functional potential of this haplogroup defining mutations. During our sequence analysis of haplogroup N1b we noticed that the Ash-specific sub-haplogroup N1b1 harbors an amino acid substitution in mtDNA position 4917 (Figure 2), which also defines haplogroup T that was previously associated with reduced sperm motility [31]. Interestingly, our permutation test suggests with borderline significance that haplogroup T might be in association (p = 0.059) with some T2DM complications in the Seph population. Previously [30,36] we showed that this mutation alters a highly conserved amino acid in the ND2 gene, hence suggesting a functional potential. Since haplogroups N1b1 and T stem from very different branches in the human mtDNA phylogeny it can be concluded that the 4917 mutation was established at least twice during human evolution. All these evidence imply that the change at position 4917 contributes to the protective effect against certain T2DM complications.

Along with haplogroup J1 association with other multi-factorial phenotypes [30,37], and its effect on the penetrance of mutations causing the eye disorder LHON [38] our results support the premise that mutations defining this haplogroup affect OXPHOS. Similar to haplogroup N1b1, some mutations defining haplogroup J1 alter amino-acids with high conservation degree: (1) a transition in position 10398 which is a Thr114Ala replacement in the ND3 subunit of complex I, shown to alter mitochondrial matrix pH in cell-culture experiments [31]; and (2) a transition in position 13708, causing a Ala458Thr replacement in the ND5 complex I subunit. In addition, haplogroup J1 harbors the G3010A substitution located within the 12SrRNA gene. Although this mutation has been found in several haplogroups [39], it is possible that the combination of this mutation with the mutations that generally define haplogroup J underlies the phenotypic effect of J1 in the Ash population.

Interestingly, the non-synonymous changes in haplogroups J1 and N1b1 altered highly conserved amino-acid positions in OXPHOS complex I, implying that they possibly affect the activity of this complex [40]. Accordingly, anti-diabetic agents (metformin and thiazolidinediones) act specifically through the inhibition of complex I activity [41], which suggests a role for complex I functional alteration in the etiology of T2DM.

## Conclusion

In summary, our results revealed notable differences in mtDNA genetic diversity within Jews. Our association study of mtDNA genetic variants with T2DM complications showed, that the differences in haplogroup distribution in the three studied populations were associated with differences in disease susceptibility factors. These findings supported our working hypothesis that minor alleles overlooked in large scale association studies may reveal their functional potential in genetic isolates.

## Methods

### Patient population

The Israeli Diabetes Research Group (IDRG) collected Jewish unrelated T2DM patients of Ashkenazi (Ash) origin (n = 762), of European non-Ashkenazi (Seph) origin (n = 191) and North African Jews (NAF) from seven medical centers in Israel. The Ashkenazi Jews belong to a relatively young population that has gone through a recent bottleneck and thus has less genetic heterogeneity than the general Caucasian population [41]. The Seph and NAF Jewish populations are as young as the Ashkenazi population, yet may have gone through different bottleneck events thus the three populations were analyzed separately. The countries of origin of the patients included in this study can be viewed in Additional file 1-methods. To avoid population stratification effects on the genetic variability, samples in the compared groups were matched for the maternal country of origin.

The basic clinical characteristics of the patients are shown in Table 4. Patients with at least 10 years of known diabetes were selected to assure a sufficiently high prevalence of diabetic complications to provide adequate statistical power in populations of this size. Using information from the patients' medical records and from structured interviews; we initially classified the patients into two groups, those who did not develop any complications after at least 10 years of clinical disease, and those who developed at least one complication. The latter group was further divided into three groups according to the complication diagnosed: retinopathy – patients with proliferating diabetic retinopathy, macular edema and/or blindness; cardiovascular disease – patients with a history of percutaneous transluminal coronary angioplasty, coronary artery bypass graft, myocardial infarction, and congestive heart failure; and nephropathy – patients with microalbuminuria (>30 but < 300 mg protein per gram of creatinine) or proteinuria (>300 mg protein per gram of creatinine), with or without decreased renal function.

DNA was extracted from peripheral lymphocytes by standard techniques (Puregene, Gentra Systems, Minneapolis, MN). Written informed consent was obtained from all individuals who participated in this study, which was approved by the Hadassah Medical Organization's Institutional Review Board for Human Studies.

### Classification of haplogroups

Genotyping was conducted by a hierarchical approach, starting from the most prevalent haplogroups in this population [42]. For detailed information see Additional file 1 – methods and Additional file 1 – Tables 7,8.

### Statistical analysis

To avoid small sample sizes, some of the haplogroups were grouped following phylogenetic considerations (for details, see Additional file 1- methods). Statistical analyses were performed using Systat 11.0 (Systat Software, Inc., CA, USA). We first used R × C (rows × columns) test of independence to compare haplogroup distribution among the three different Jewish populations. Next we used a permutation test to detect candidate haplogroups with altered representation in the complication groups in each of the three Jewish populations. Permutation tests were performed using a MATLAB (v.6.5) script: Complications (a binary indicator variable, 0 – no complication and 1 – complication present) were randomly assigned without replacement to patients with different mtDNA genetic backgrounds (i.e., haplogroups). The proportion of patients in each haplogroup who developed a specific complication was calculated. Next the absolute difference (two-tailed test) between each of these values and general tendency to develop such a complication in the entire population (i.e., the proportion of patients in the population who developed such a complication irrespective of their genetic background) was recorded. This procedure was repeated 10,000 times. P values were estimated as the proportion cases in which the absolute difference obtained during the simulations was equal to or greater than that of the original data set. Finally, to test whether the susceptibility to develop T2DM complications (represented by a binary indicator variable taking on values 0 and 1) differed among haplogroups, logistic regression was performed to adjust for patient characteristics, i.e., disease duration, sex and age. It is notable that by converting the categorical variable "haplogroup" into a dummy variable, we could compare the candidate haplogroups with each of the other haplogroups using a single test, i.e., to avoid multiple testing. Specifically, since this variable composed of 8–10 classes (depending on the population analyzed), its inclusion in a logistic regression model requires generating 7–9 indicator variables, respectively. The coefficients of these indicator variables indicate whether the propensity to develop complications in each of the respective haplogroups differs from that of the reference (candidate) haplogroup (haplogroup J1 or N1b in the Ash and the HV* lineage in the NAF populations). For simplicity, we have presented in the text results from analyses in which we treated all the haplogroups in the aggregate excluding the reference haplogroup. The complete analyses, in which these candidate haplogroups were compared with each of the other haplogroups, are presented in Additional file 1 – Tables 2-4. To obtain an estimate for the relative risk of carriers of a particular haplogroup to develop T2DM complications, odds ratios (ORs) were calculated. Power analysis was conducted to get an estimate of the sample size required to replicate our results (see discussion). Although it was argued in the past that corrections are not necessary in our case [43], we have considered the three different complications examined as potential multiple testing and the statistical significance was Bonferonni corrected to α<0.017.

### Whole mtDNA sequencing

The mtDNA genome of normal non-T2DM individuals was amplified in 3 overlapping DNA fragments, and was sequenced using the mitochondrial DNA re-sequencing chip, as previously described [44] ; sequence ambiguities were resolved using specific primers with a conventional ABI 3100 sequencer. For details see Additional file 1 – methods. Changes within the sequences as compared with the Cambridge reference sequence were documented (see Additional file 1 – Table 5).

### Phylogenetic reconstruction

Sequence alignment, phylogenetic reconstruction and bootstrap analysis were performed with MEGA3 software [31].

### Estimating degrees of conservation of variants in the mtDNA

Alignment of each of the mtDNA encoded proteins and rRNA genes in 40 vertebrates (including man) and two invertebrates (*Drosophila *and octopus) was performed (see Additional file 1 – Table 5,6). This alignment was used to assess the degree of conservation (CD), i.e., the number of species that shared the same exact amino acid or nucleotide positions in protein-coding or rRNA-coding mtDNA genes, respectively (see Additional file 1 – Table 6). The CD of the naturally occurring variants in the whole mtDNA sequences was compared with the mean CD of 20 mtDNA disease-causing mutations (36 ± 9) as previously described. A position was considered highly conserved if its CD was within one standard deviation from the mean CD of the disease-causing mutations.

## Authors' contributions

JF performed the research and analyzed the data; IB, SA and SD analyzed the data. OO analyzed the data and assisted in the design of the statistical analyses. JW and IR collected the data. DEA performed sequence analysis. BG assisted in the design of research and collected the data. DM designed the research and wrote the paper.

## Supplementary Material

Additional File 1 The file includes additional methods and additional tables that are referred to in the text. Click here for file
