# Supplementary material for: Differences in mtDNA haplogroup distribution among 3 Jewish populations alter susceptibility to T2DM complications
Source: BMC Genomics. 2008 Apr 29;9:198. doi: 10.1186/1471-2164-9-198 (PMC2386827; doi:10.1186/1471-2164-9-198)
Supplement: Additional File 1 — The file includes additional methods and additional tables that are referred to in the text. [file 1471-2164-9-198-S1.doc]

**Additional file 1: methods**

**Patients' counties of origin:** In the cohorts included in this study the patients originate from the following countries; Ash cohort – Germany, Austria, Switzerland, former Czechoslovakia, Hungary, Yugoslavia, Israel, United Kingdom, Ireland, Poland, USA, Canada, Holland, Argentina, Ukraine, Russia, Latvia, Lithuania and Romania; Seph cohort – Cyprus, Israel, Bulgaria, Greece, Spain, Italy and Turkey; NAF cohort – Morocco, Algeria, Tunisia, Libya and Egypt. Since Jews from all three types of cohorts live in Israel, when the country of origin of the patients was "Israel", they were assigned to the relevant cohort on the basis of their declared origin—Ash, Seph or NAF.

**Classification of haplogroups:** Genotyping was conducted by a hierarchical approach, starting from the most prevalent lineages in the European population, U and HV lineages, followed by haplogroups K1, K2, H, N1b, J1, J2 and T, and then the remainder of the less prevalent haplogroups in this population ^1^. Haplogroup N1b classification was confirmed by mtDNA HVR1 sequencing. Supplementary Tables 7 and 8 summarize the list of single nucleotide repeats (SNPs), primers, restriction enzymes, and PCR conditions.

**Additional file 1- statistics**: To avoid small sample sizes, some of the haplogroups were grouped following phylogenetic considerations. Accordingly, I, W and X were grouped in all cohorts. Similar to previous population analysis of Ashkenazi Jews ^1^, among the 61 Ash patients belonging to haplogroup J, only two were J2, while the majority belonged to haplogroup J1. This uneven distribution of J1 and J2 patients within haplogroup J, led to excluding the J2 patients from the analyses, and the group was defined as J1. Due to small sample size of haplogroup J2 in the Seph and NAF cohorts, the statistical analyses were performed only on haplogroup J1 in these cohorts as well. Haplogroups JT* (R2) and L were clustered together with the unknown haplogroups in the "other" group and haplogroup V was clustered together with HV^*^ in all three populations.

To avoid decrease in the power of the study other variables such as medications and smoking were not included in the analysis (even though such data are available to us); this was done since such variables are likely to be strongly influenced by the presence of complications per se, e.g., patients with nephropathy are more likely to be taking angiotensin converting enzyme (ACE) inhibitors, and patients with cardiovascular disease are more likely to be under treatment with statins and to have stopped smoking. **Power analysis** was conducted to estimate the population sizes required to replicate the results obtained in this study.

**Screening the patient cohort for the mtDNA A3243G mutation***.* Patients included in the Ash cohort (initially, n = 765) were screened for the A3243G mtDNA mutation, which occur in ~1% of T2DM patients in various populations. The screening was performed as previously described ^2^. Three individuals were detected having the mutation and were excluded from the study. Since there is no supported association of this mutation with a specific haplogroup ^3^, we decided not to continue screening for this mutation in the non-Ashkenazi Jewish populations.

**Whole mtDNA sequencing**. The mtDNA genome of normal non-T2DM individuals was amplified using three primer pairs, with Phusion Taq polymerase (Finnzymes®) under the following conditions: 2 min 94 ºC 30×(94 oC 15 s, 68 ºC, 7 min), 12 min 68 ºC, 4 ºC. The following primers were used:

Fragment 1(forward) – 5'-ATAGGGGTCCCTTGACCACCATCCTCCGT-3'

Fragment 1(reverse) – 5'- GAGCTGTGCCTAGGACTCCAGCTCATGCGCCG-3'

Fragment 2(forward) – 5'-CGGCCTGCTTCTTCTCACATGACAAAAAC-3'

Fragment 2(reverse) – 5'- GATCAGGAGAACGTGGTTACTAGCACAGAGAG-3'

Fragment 3(forward) – 5' CATTCTCATAATCGCCCACGGGCTTACATCC-3'

Fragment 3(reverse) – 5'- GTTCGCCTGTAATATTGAACGTAGGTGCG-3'

Table 1 (Additional file): **P-values** obtained from the permutation test.

| **Haplogroup** | **Cardiovascular** | | | **Retinopathy** | | | **Nephropathy** | | |
| --- | --- | --- | --- | --- | --- | --- | --- | --- | --- |
|  | **Ash,** | **Seph** | **NAF** | **Ash** | **Seph** | **NAF** | **Ash** | **Seph** | **NAF** |
| U (nonK) | *0.27* | 0.72 | 0.76 | *1* | 0.41 | 0.22 | *1* | 1 | 0.6 |
| K (K1-Ash) | *0.41* | 0.58 | 0.19 | *1* | 0.22 | 1 | *0.28* | 0.59 | 0.35 |
| K2 (Ash) | *0.71* | --- | --- | *0.66* | --- | --- | *1* | --- | --- |
| ^1^HV^*^ | *0.89* | 0.73 | **0.01** | *0.58* | **0.05** | 0.075 | *0.56* | 0.16 | **0.02** |
| H | *0.41* | 0.84 | 0.18 | *0.52* | 0.33 | 0.26 | *0.73* | 0.55 | 0.4 |
| J1 | 0.46 | 0.73 | 0.4 | **0.035** | 0.72 | 0.064 | **0.02** | 0.4 | 0.48 |
| T | 0.49 | 0.45 | 0.41 | 0.41 | 0.41 | 0.38 | 0.37 | 0.059 | 0.41 |
| N1b | 0.12 | 1 | --- | 0.3 | 0.62 | --- | **0.003** | 1 | --- |
| IWX | 0.49 | 1 | 0.55 | 0.24 | 1 | 0.38 | 0.7 | 0.72 | 0.61 |
| ^2^Others | 0.78 | 1 | 0.37 | 0.34 | 0.37 | 0.79 | 0.39 | 0.81 | 1 |
| **Total** | **261** | | | **118** | | | **308** | | |

^1^HV^*^ - see table 1a. ^2^Others – see table 1a. ^**^After removing the J2 patients (see Materials and Methods).

Table 2 (Additional file): **Ash population** - Logistic regression analyses testing for differences in the propensity to develop complications (nephropathy, retinopathy and cardiovascular) between patients pertaining to haplogroup N1b and those in each of the other 9 haplogroups. In order to control for the possible effects of age, gender and disease duration on the propensity to develop complications all three variables were included in the logistic regression model.

| Haplogroup | Nephropathy vs. no complication | | Retinopathy vs. no complication | | Cardiovascular vs. no complication | |
| --- | --- | --- | --- | --- | --- | --- |
|  | P-Value | OR  (95% CI) | P-Value | OR  (95% CI) | P-Value | OR  (95% CI) |
| H | ***0.015*** | ***2.8***  ***(1.22-6.57)*** | 0.223 | 2  (0.66-6.13) | ***0.022*** | ***2.7***  ***(1.15-6.25)*** |
| HV* | **0.088** | **2.28**  **(0.89-5.9)** | 0.209 | 2.2  (0.64-7.5) | **0.06** | **2.49**  **(0.96-6.44)** |
| K1 | ***0.004*** | ***3.5***  ***(1.5-8.11)*** | 0.204 | 2.1  (0.67-6.6) | ***0.012*** | ***2.97***  ***(1.27-6.96)*** |
| K2 | ***0.028*** | ***3.14***  ***(1.13-8.7)*** | 0.488 | 1.67  (0.4-7.1) | **0.072** | **2.7**  **(0.92-7.94)** |
| J1 | ***0.002*** | ***5***  ***(1.83-13.7)*** | ***0.041*** | ***3.9***  ***(1.1-14.3)*** | ***0.046*** | ***2.9***  ***(1.02-8.4)*** |
| T | 0.14 | 2.3  (0.77-7.03) | 0.806 | 0.81  (0.15-4.32) | 0.312 | 1.8  (0.56-6.1) |
| U (non K) | ***0.045*** | ***2.7***  ***(1.02-7.3)*** | 0.71 | 1.3  (0.33-5.2) | 0.433 | 1.5  (0.53-4.47) |
| IWX | ***0.042*** | ***3.1***  ***(1.04-9.05)*** | 0.643 | 0.64  (0.1-4.2) | 0.309 | 1.87  (0.56-6.3) |
| OTHER | 0.13 | 2.1  (0.8-5.5) | 0.732 | 1.3  (0.34-4.7) | ***0.026*** | ***2.93***  ***(1.14-7.54)*** |

Table 3 (Additional file): **Ash population** -Logistic regression analyses testing for differences in the propensity to develop complications (nephropathy, retinopathy and cardiovascular) between patients pertaining to haplogroup J1 and those in each of the other 9 haplogroups. Note that analyses were done on groups of T2DM patients with nephropathy or retinopathy, both excluding cardiovascular disease, and on T2DM patients with heart disease that did not develop microvascular complications (i.e., without nephropathy and retinopathy). In order to control for the possible effects of age, gender and disease duration on the propensity to develop complications all three variables were included in the logistic regression model.

| Haplogroup | Nephropathy NO Cardiovascular vs. no complication | | Retinopathy NO cardiovascular vs. no complication | | Cardiovascular without microvascular complications vs. no complication | |
| --- | --- | --- | --- | --- | --- | --- |
|  | P-Value | OR  (95% CI) | P-Value | OR  (95% CI) | P-Value | OR  (95% CI) |
| H | ***0.036*** | ***0.43***  ***(0.197-.095)*** | **0.067** | **0.37**  **(0.13-1.1)** | 0.84 | 1.11  (0.39-3.2) |
| HV* | ***0.045*** | ***0.39***  ***(0.15-0.98)*** | 0.14 | 0.39  (0.11-1.3) | 0.67 | 1.29  (0.4-4.1) |
| K1 | 0.15 | 0.56  (0.26-1.2) | 0.15 | 0.45  (0.16-1.3) | 0.55 | 1.38  (0.49-3.9) |
| K2 | 0.14 | 0.46  (0.17-1.3) | 0.18 | 0.35  (0.07-1.6) | 0.96 | 1.04  (0.26-4.1) |
| N1b | ***0.004*** | ***0.22***  ***(0.074-0.62)*** | ***0.034*** | ***0.18***  ***(0.04-0.88)*** | 0.67 | 0.76  (0.22-2.6) |
| T | 0.12 | 0.42  (0.14-1.25) | ***0.035*** | ***0.087***  ***(0.009-0.8)*** | 0.78 | 1.2  (0.31-4.8) |
| U (non K) | 0.21 | 0.54  (0.21-1.4) | **0.07** | **0.26**  **(0.06-1.1)** | 0.93 | 0.94  (0.26-3.4) |
| IWX | 0.22 | 0.51  (0.17-1.5) | ***0.046*** | ***0.1***  ***(0.01-0.96)*** | 0.31 | 0.39  (0.07-2.4) |
| OTHER | ***0.007*** | ***0.25***  ***(0.09-0.69)*** | **0.08** | **0.32**  **(0.087-1.1)** | 0.56 | 1.41  (0.44-4.5) |

Table 4 (Additional file): **NAF population** - Logistic regression analyses testing for differences in the propensity to develop complications (nephropathy, retinopathy and cardiovascular) between patients pertaining to haplogroup aggregate HV^*^ and those in each of the other 7 haplogroups. In order to control for the possible effects of age, gender and disease duration on the propensity to develop complications all three variables were included in the logistic regression model.

| Haplogroup | Nephropathy vs. no complication | | Retinopathy vs. no complication | | Cardiovascular vs. no complication | |
| --- | --- | --- | --- | --- | --- | --- |
|  | P-Value | OR  (95% CI) | P-Value | OR  (95% CI) | P-Value | OR  (95% CI) |
| H | 0.17 | 0.45  (0.15-1.4) | 0.3 | 0.52  (0.14-1.8) | 0.14 | 0.39  (0.12-1.3) |
| T | **0.064** | **0.15**  **(0.019-1.1)** | **0.061** | **0.091**  **(0.007-1.1)** | **0.074** | **0.11**  **(0.009-1.2)** |
| K | ***0.037*** | ***0.25***  ***(0.068-0.92)*** | 0.19 | 0.38  (0.09-1.6) | ***0.02*** | ***0.15***  ***(0.03-0.74)*** |
| J1 | **0.055** | **0.18**  **(0.03-1.04)** | 0.98 | 0 | ***0.048*** | ***0.82***  ***(0.007-0.98)*** |
| U (non K) | ***0.045*** | ***0.24***  ***(0.06-0.97)*** | ***0.019*** | ***0.11***  ***(0.017-0.7)*** | ***0.043*** | ***0.19***  ***(0.039-0.95)*** |
| IWX | **0.057** | **0.27**  **(0.068-1.04)** | ***0.027*** | ***0.14***  ***(0.024-0.8)*** | ***0.033*** | ***0.17***  ***(0.03-0.87)*** |
| OTHER | 0.17 | 0.39  (0.1-1.5) | 0.32 | 0.46  (0.1-2.1) | **0.093** | **0.23**  **(0.04-1.3)** |

Additional file - Table 5: Record of nucleotide changes versus the Cambridge reference sequence in all of the whole mtDNA N1b sequences included in this study. Conservation degree: The nominator is the number of species harboring identical nucleotide or amino-acid position out of 42 vertebrates and invertebrates compared mtDNA gene sequences (see methods section and supplementary table 4).

| **Position** | **Nucleotide change** | **Access number of sequences with the change** | **Type of Change** | **Conservation Degree** |
| --- | --- | --- | --- | --- |
| 73 | A 🡪 G | All | Non-coding |  |
| 151 | C 🡪 T | Singleton 79_Ash | Non-coding |  |
| 152 | T 🡪 C | All | Non-coding |  |
| 185 | G 🡪 A | Singleton 34002_Pal | Non-coding |  |
| 188 | A 🡪 G | Singleton 34002_Pal | Non-coding |  |
| 263 | A 🡪 G | All | Non-coding |  |
| 303 | Insertion of C | 12 individuals (9 Ash; 3 Pal) | Non-coding |  |
| 311 | Insertion of C | All except 89_Ash | Non-coding |  |
| 379 | A 🡪 G | Singleton 89_Ash | Non-coding |  |
| 452 | Insertion of T | 505002_Pal  4002_Pal | Non-coding |  |
| 514 | Deletion of C | gi|17985627| | Non-coding |  |
| 515 | Deletion of A | gi|17985627| | Non-coding |  |
| 550 | Deletion of A | 2164_Ash | Non-coding |  |
| 563 | A 🡪 G | Singleton 89_Ash | Non-coding |  |
| 750 | A 🡪 G | All | Inside 12SrRNA | 36/42 |
| 1438 | A 🡪 G | All | Inside 12SrRNA | 37/42 |
| 1598 | G 🡪 A | All | Inside 12SrRNA | 42/42 |
| 1703 | C 🡪 T | All, except 4002_Pal and 505002_Pal | Inside 16SrRNA | 14/42 |
| 1719 | G 🡪 A | All | Inside 16SrRNA | 35/42 |
| 2639 | C 🡪 T | All | Inside 16SrRNA | 25/42 |
| 2706 | A 🡪 G | All | Inside 16SrRNA | 30/42 |
| 3580 | Deletion of C | 8002_Pal | Inside 16SrRNA | 42/42 |
| 3921 | C 🡪 A | All | Syn (ND1) | 42/42 |
| 4735 | C 🡪 A | All except all Pal + gi|17985627| | nSyn (T89NND2) | 4/42 |
| 4769 | A 🡪 G | All | Syn (ND2) | 12/42 |
| 4904 | C 🡪 T | Singleton 8002_Pal | Syn(ND2) | 13/42 |
| 4917 | A 🡪 G | All except all Pal + gi|17985627| | nSyn (N150DND2) | 38/42 |
| 4960 | C 🡪 T | All | nSyn (A164VND2) | 6/42 |
| 5471 | G 🡪 A | All | Syn (ND2) | 26/42 |
| 5528 | T 🡪 C | Singleton 34002_Pal |  |  |
| 6045 | C 🡪 T | Singleton 34002_Pal | nSyn (L47FCOI) | 41/42 |
| 7028 | C 🡪 T | All | Syn (COI) | 42/42 |
| 7526 | A 🡪 T | Singleton 123_Ash |  |  |
| 8020 | G 🡪 A | 34002_Pal and 37002_Pal | Syn (COII) | 41/42 |
| 8084 | A 🡪 G | 505002_Pal and 4002_Pal | nSyn (T167ACOII) | 8/42 |
| 8251 | G 🡪 A | All | Syn (COII) | 9/42 |
| 8261 | A 🡪 G | Singleton 34002_Pal | nSyn (T226ACOII) | 9/42 |
| 8410 | C 🡪 T | Singleton 34002_Pal | Syn (ATP8) | 6/42 |
| 8472 | C 🡪 T | All except  gi|17985627| | nSyn (P36LATP8) | 10/42 |
| 8676 | C 🡪 T | Singleton 2164_Ash | Syn (ATP6) | 21/42 |
| 8763 | T 🡪 C | Singleton 34002_Pal | Syn (ATP6) | 32/42 |
| 8836 | A 🡪 G | All | nSyn (M104VATP6) | 30/42 |
| 8860 | A 🡪 G | All | nSyn(A112TATP6) | 31/42 |
| 9230 | T 🡪 C | Singleton 8002_Pal | Syn (COIII) | 38/42 |
| 9335 | C 🡪 T | All non-Ash except 8002_Pal and Herrnstadt | Syn (COIII) | 38/42 |
| 9438 | G 🡪 A | Singleton 37002_Pal | nSyn (G78SCOIII) | 37/42 |
| 9882 | C 🡪 T | Singleton 8002_Pal | nSyn (H226YCOIII) | 42/42 |
| 9957 | T 🡪 C | Singleton 33002_pal | nSyn (F251LCOIII) | 41/42 |
| 10238 | T 🡪 C | All | Syn (ND3) | 40/42 |
| 11362 | A 🡪 G | All Pal and gi|17985627| | Syn (ND4) | 30/42 |
| 11719 | G 🡪 A | All except 2130_Ash, 123_Ash and 62_Ash | Syn (ND4) | 41/42 |
| 11928 | A 🡪 G | All Ash, gi|82792304|, gi|82792542| and Herrnstadt | nSyn(N390SND4) | 26/42 |
| 12092 | C 🡪 T | All Ash, gi|82792304|, gi|82792542| and Herrnstadt | nSyn(L445FND4) | 25/42 |
| 12372 | G 🡪 A | Singleton gi|17985627| | Syn (ND5) | 23/42 |
| 12501 | G 🡪 A | All | Syn (ND5) | 5/42 |
| 12705 | C 🡪 T | All | Syn (ND5) | 14/42 |
| 12822 | A 🡪 G | All | Syn (ND5) | 12/42 |
| 12891 | C 🡪 T | Singleton 37002_Pal | Syn (ND5) | 23/42 |
| 13114 | C 🡪 A | Singleton BGU_123 | nSyn (L260IND5) | 40/42 |
| 13129 | C 🡪 T | All Ash, gi|82792304|, gi|82792542| and Herrnstadt | nSyn (P265SND5) | 39/42 |
| 13608 | T 🡪 C | Singleton 8002_Pal | (ND5) | 23/42 |
| 13635 | T 🡪 C | Singleton 79_Ash | Syn (ND5) | 30/42 |
| 13710 | A 🡪 G | All Ash, gi|82792304|, gi|82792542| and Herrnstadt | Syn (ND5) | 20/42 |
| 13768 | T 🡪 C | Singleton 37002_Pal | nSyn (F478LND5) | 2/42 |
| 14581 | T 🡪 C | All Ash, gi|82792304|, gi|82792542| and Herrnstadt | Syn (ND6) | 30/42 |
| 14766 | C 🡪 T | All | nSyn(I7Tcytb) | 3/42 |
| 15043 | G 🡪 A | Singleton 8002_Pal | Syn (cytb) | 38/42 |
| 15071 | T 🡪 C | Singleton 37002_Pal | nSyn (Y109Hcytb) | 13/42 |
| 15079 | A 🡪 G | Singleton 37002_Pal | Syn (cytb) | 37/42 |
| 15326 | A 🡪 G | All | nSyn (A194Tcytb) | 8/42 |
| 15883 | G 🡪 A | Singleton 8002_Pal | Syn (cytb) | 37/42 |
| 16037 | A 🡪 G | Singleton 4002_Pal | Non-coding |  |
| 16075 | T 🡪 C | Singleton 4002_Pal | Non-coding |  |
| 16093 | T 🡪 C | Singleton 37002_Pal | Non-coding |  |
| 16129 | G 🡪 A | Singletons 34002_Pal and 37002_Pal | Non-coding |  |
| 16145 | G 🡪 A | All except 505002_Pal | Non-coding |  |
| 16176 | C 🡪 G | gi|17985627| and all Pal except 505002_Pal | Non-coding |  |
| 16176 | C 🡪 A | All Ash, gi|82792304| and gi|82792542| | Non-coding |  |
| 16180 | A 🡪 G | Singleton gi|17985627| | Non-coding |  |
| 16223 | C 🡪 T | All except 505002_Pal | Non-coding |  |
| 16256 | C 🡪 T | Singleton 33002_Pal | Non-coding |  |
| 16291 | C 🡪 T | Singleton 34002_Pal | Non-coding |  |
| 16311 | T 🡪 C | Singleton 4002_pal | Non-coding |  |
| 16390 | G 🡪 A | All except 89_Ash and 505002_Pal | Non-coding |  |
| 16519 | T 🡪 C | All except 34002_Pal | Non-coding |  |

Additional file - Table 6: Accession numbers of mtDNA sequences of species included in the multiple sequence alignments.

Additional file - Table 7: Assayed SNPs, Primers and PCR conditions for mtDNA haplogroup analysis.

| **Species** | **Accession number** |
| --- | --- |
| Homo sapiens mitochondrion | NC_001807 |
| Pan paniscus mitochondrion | NC_001644 |
| Pan troglodytes mitochondrion | NC_001643 |
| Gorilla gorilla mitochondrion | NC_001645 |
| Pongo pygmaeus abelii mitochondrion | NC_002083 |
| Hylobates lar mitochondrion | NC_002082 |
| Macaca mulatta mitochondrion | NC_005943 |
| Colobus guereza mitochondrion | NC_006901 |
| Lemur catta mitochondrion | NC_004025 |
| Loxodonta africana mitochondrion | NC_000934 |
| Canis familiaris mitochondrion | NC_002008 |
| Felis catus mitochondrion | NC_001700 |
| Bos taurus mitochondrion | NC_006853 |
| Sus scrofa mitochondrion | NC_000845 |
| Equus caballus mitochondrion | NC_001640 |
| Capra hircus mitochondrion | NC_005044 |
| Mus musculus mitochondrion | NC_005089 |
| Rattus norvegicus mitochondrion | NC_001665 |
| Ornithorhynchus anatinus mitochondrion | NC_000891 |
| Gallus gallus mitochondrion | NC_001323 |
| Danio rerio mitochondrion | NC_002333 |
| Drosophila melanogaster mitochondrion | NC_001709 |
| Crocodylus niloticus mitochondrion | NC_008142 |
| Balaenoptera musculus mitochondrion | NC_001601 |
| Cavia porcellus mitochondrion | NC_000884 |
| Cebus albifrons mitochondrion | NC_002763 |
| Salmo salar mitochondrion | NC_001960 |
| Tarsius bancanus mitochondrion | NC_002811 |
| Ursus americanus mitochondrion | NC_003426 |
| Xenopus laevis mitochondrion | NC_001573 |
| Rana nigromaculata mitochondrion | NC_002805 |
| Python regius mitochondrion | NC_007399 |
| Papio hamadryas mitochondrion | NC_001992 |
| Ovis aries mitochondrion | NC_001941 |
| Oryzias latipes mitochondrion | NC_004387 |
| Octopus vulgaris mitochondrion | NC_006353 |
| Iguana iguana mitochondrion | NC_002793 |
| Lama pacos mitochondrion | NC_002504 |
| Cricetulus griseus mitochondrion | NC_007936 |
| Oncorhynchus mykiss mitochondrion | NC_001717 |
| Phoca vitulina mitochondrion | NC_001325 |
| Macropus robustus mitochondrion | NC_001794 |

| **Haplogroup**  **& Polymorphic Site** | MgCl_2_ conc. & annealing temp. | **Primer Sequences (5’ – 3’)** |
| --- | --- | --- |
| U  +12308 (*HinfI*) | 2mM MgCl  Annealing temp: 55ºC | F: ACGACCCCTTATTTACCGAG  R:ATTACTTTTATTTGGAGTTGCACCAAGATT |
| HV  -14766 (*MseI*) | 1.5mM MgCl  Annealing temp: 55ºC | F: CTAAACCCCCATAAATAGGAG  R: GGGAGGTCGATGAATGAGTG |
| H  -7025 (*AluI*) | 2mM MgCl  Annealing temp: 55ºC | F: CACTCCACGGAAGCAATATG  R: GGATTTTGGCGTAGGTTTGG |
| K  -9052 (*HaeII*) | 2mM MgCl  Annealing temp: 55ºC | F: AGCCCACTTCTTACCACAAG  R: TAGGCTTGGATTAAGGCGAC |
| K2  +9216 *(HaeIII)* | 2mM MgCl  Annealing temp: 58ºC | F: TCCCACTCCTAAACACATCC  R: GCCAATAATGACGTGAAGTCC |
| JT  +4216 (*NlaIII*) | 1.5mM MgCl  Annealing temp: 55ºC | F: ATACCCCCGATTCCGCTACG  R: ATGGGGTGTGATAGGTGGC |
| J  -13704 (*BstNI*) | 1.5mMx MgCl  Annealing temp: 55ºC | F: TCGAATAATTCTTCTCACCC  R: TAGTAATGAGAAATCCTGCG |
| J2  -7475 (*AluI*) | 2mM MgCl  Annealing temp: 58ºC | F: GATTTGAGAAGCCTTCGCTTC  R: TTTGAAAAAGTCATGGAGGCC |
| T  +15606 (*AluI*) | 1.5mM MgCl  Annealing temp: 58ºC | F: ACCTTCCACCCTTACTACAC  R: GCGGCTAGGAGTCAATAAAG |
| N1  +10237 (*HphI*) | 1.5mM MgCl  Annealing temp:59ºC | F: TTACGAGTGCGGCTTCGACC  R: ACTCATAGGCCAGACTTAGG |
| I  +10032(*AluI*) | 1.5mM MgCl  Annealing temp: 55ºC | F: CTCCATCTATTGATGAGGGTC  R: CTCGTAAGGGGTGGATTTTTC |
| W  -8994(*HaeIII*) | 1.5x MgCl  Annealing temp: 55ºC | F: CTATAAACCTAGCCATGGCC  R: TAGGCTTGGATTAAGGCGAC |
| X  +14465(*AccI*) | 1.5mM MgCl  Annealing temp: 55ºC | F: ATCCTACCTCCATCGCTAAC  R: GCCTTCTCCTATTTATGGGG |

Additional file - Table 8: SNPs (Single Nucleotide Polymorphisms) used for mtDNA haplogroup analysis. Haplogroup analysis was performed in a hierarchical manner starting from the most prevalent lineages in Ashkenazi Jews (U and the HV lineage). (N) Site not analyzed in the particular haplogroup. Restriction site were present (+) or lost (-) in samples carrying the studied haplogroup. All samples belonging to N1 (non I) haplogroup were subjected to HVR1 sequence revealing a common 16145A-16176A-16223T-16390A motif as previously described in Ashkenazi N1b ^1^.

| Haplogroup | SNP nucleotide position in the mtDNA | | | | | | | | | | | | | |
| --- | --- | --- | --- | --- | --- | --- | --- | --- | --- | --- | --- | --- | --- | --- |
|  | 1715 Dde1 | 4216 NlaIII | 7025 Alu1 | 8994 HaeIII | 7445  Alu1 | 9052  HaeII | 9216  HaeIII | 10032  Alu1 | 10237 HphI | 12308 Hinf1 | 13704 BstNI | 14465  AccI | 14766  MseI | 15606 Alu1 |
| U | N | N | N | N | N | + | N | N | N | + | N | N | N | N |
| K | N | N | N | N | N | - | N | N | N | + | N | N | N | N |
| K1 | N | N | N | N | N | - | - | N | N | + | N | N | N | N |
| K2 | N | N | N | N | N | - | + | N | N | + | N | N | N | N |
| HV | N | N | N | N | N | + | N | N | N | - | N | N | - | N |
| HV* | N | N | + | N | N | + | N | N | N | - | N | N | - | N |
| H | N | N | - | N | N | + | N | N | N | - | N | N | - | N |
| JT | N | + | + | N | N | + | N | N | N | - | N | N | + | N |
| J | N | + | + | N | N | + | N | N | N | - | - | N | + | N |
| J1 | N | + | + | N | + | + | N | N | N | - | - | N | + | N |
| J2 | N | + | + | N | - | + | N | N | N | - | - | N | + | N |
| T | N | + | + | N | N | + | N | N | N | - | + | N | + | + |
| N1IX | - | - | + | N | N | + | N | N | N | - | + | N | + | - |
| N1(non I) | - | - | + | N | N | + | N | N | + | - | + | N | + | - |
| I | - | - | + | N | N | + | N | + | - | - | + | N | + | - |
| X | - | + | + | N | N | + | N | - | - | - | + | + | + | - |
| W | + | + | + | - | N | + | N | - | - | - | + | - | + | - |

**References**

1 Behar DM, Hammer MF, Garrigan D *et al*: MtDNA evidence for a genetic bottleneck in the early history of the Ashkenazi Jewish population. *Eur J Hum Genet* 2004; **12**: 355-364.

2 Ohkubo K, Yamano A, Nagashima M *et al*: Mitochondrial gene mutations in the tRNA(Leu(UUR)) region and diabetes: prevalence and clinical phenotypes in Japan. *Clin Chem* 2001; **47**: 1641-1648.

3 Torroni A, Campos Y, Rengo C *et al*: Mitochondrial DNA haplogroups do not play a role in the variable phenotypic presentation of the A3243G mutation. *Am J Hum Genet* 2003; **72**: 1005-1012.
